# The Association between Alcohol Hangover Frequency and Severity: Evidence for Reverse Tolerance?

**DOI:** 10.3390/jcm8101520

**Published:** 2019-09-21

**Authors:** Joris C. Verster, Karin A. Slot, Lizanne Arnoldy, Albertine E. van Lawick van Pabst, Aurora J. A. E. van de Loo, Sarah Benson, Andrew Scholey

**Affiliations:** 1 Division of Pharmacology, Utrecht Institute for Pharmaceutical Sciences (UIPS), Utrecht University, 3584CG Utrecht, The Netherlands; 2 Institute for Risk Assessment Sciences (IRAS), Utrecht University, 3584CM Utrecht, The Netherlands; 3 Centre for Human Psychopharmacology, Swinburne University, VIC 3122 Melbourne, Australia

**Keywords:** alcohol, hangover, frequency, severity, tolerance

## Abstract

Although hangover is a common consequence of heavy alcohol consumption, the area is heavily under-researched. Hangover frequency is a potential predictor of future alcohol use disorder that may be affected by hangover severity, yet the relationship between hangover frequency and severity has not been investigated. Using different methodologies and assessment instruments, two surveys, and one naturalistic study collected data on hangover frequency, hangover severity, and alcohol consumption. The relationship between hangover frequency and severity was investigated via correlational analysis, considering potentially moderating variables including alcohol intake, estimated blood alcohol concentration, demographics, and personality characteristics. In all the three studies, a positive and significant association between hangover frequency and severity was found, which remained significant after correcting for alcohol intake and other moderating factors. These findings suggest that hangover severity increases when hangovers are experienced more frequently and may be driven by sensitization or reverse tolerance to this aspect of alcohol consumption. Future research should further investigate the relationship between hangover frequency and severity and alcohol use disorder and its implications for prevention.

## 1. Introduction

Alcohol hangover refers to the combination of mental and physical symptoms, experienced the day after a single episode of heavy drinking, starting when the blood alcohol concentration (BAC) approaches zero [1]. Although hangover is a common consequence of heavy alcohol consumption, research investigating hangover frequency is sparse [2], and perhaps surprisingly, the relationship between hangover frequency and hangover severity has not been investigated. 

This is potentially important, as experiencing frequent hangovers has been associated with significant health consequences and mortality. For example, there is a significant positive association between hangover frequency and cardiovascular mortality [3]. A negative relationship has also been identified between intelligence quotient (IQ) at age 11 and hangover frequency in middle-age [4]. Whereas childhood socioeconomic status had no relevant influence, the association between childhood IQ and later life hangover frequency was significantly attenuated by middle age socioeconomic status. Other reports have identified a significant relationship between hangover frequency and experiencing depressive symptoms [5,6]. These studies did not explore the relationship between hangover frequency and hangover severity. The latter is important as it has been suggested that having hangovers frequently could be a predictor of future alcohol use disorder [7]. 

If tolerance develops when hangovers are experienced more frequently, i.e., their severity diminishes, this could feasibly lead to higher alcohol consumption. Alternatively, if hangover severity remains constant or increases with greater frequency, this may have a protective effect in that drinkers would consume less alcohol on future drinking occasions. A prospective study using the sensitivity to the effects of alcohol (SRE) scale, showed that drinkers who have a low sensitivity to alcohol (i.e., more drinks are needed to feel an effect), report hangovers less frequently following a given number of drinks [8]. These findings suggest that tolerance develops to experiencing hangovers in subjects who report frequent heavy drinking episodes. Conversely, Courtney et al. [9] found that having more frequent hangovers was a marker for increased future numbers of alcoholic drinks per drinking day. Importantly, neither study assessed hangover severity, which may play a pivotal role in the relationship between hangover frequency and drinking quantity.

Recently, Köchling et al. [10] examined the impact of the sequence of consuming beer and wine on hangover severity in 90 participants. On two test days, beer, wine, or both were consumed to reach a BAC of 0.12%. Overall hangover severity was assessed as a composite score of eight individual items, including thirst, fatigue, headache, dizziness, nausea, stomach ache, tachycardia, and loss of appetite. The intensity of these symptoms was scored on a seven-point scale, with the sum score representing the overall hangover severity ranging from 0 (absent) to 56 (extreme hangover). Hangover frequency was assessed using five categories, including ‘rarely’, ‘once-monthly’, ‘more than once-monthly, and less than once-weekly’, ‘once-weekly’, and ‘more than once-weekly’. The authors reported that hangover frequency was not a significant predictor of hangover severity. These findings suggest that hangover frequency and severity are unrelated. However, it should be considered that (1) a very crude measure of hangover frequency was used which provides little differentiation between the subjects, and (2) overall hangover severity was computed as a sum score from individual hangover symptoms, and no overall hangover severity rating was obtained. It is, thus important to further investigate the relationship between hangover frequency and severity using more precise measures.

Therefore, the current analysis investigated whether tolerance develops for experiencing alcohol hangovers. We interrogated databases from three independent studies which utilized different methodological approaches to evaluate hangover severity and frequency. It was hypothesized that hangover frequency would negatively correlate with hangover severity. In other words, if hangovers are experienced more frequently, their intensity diminishes, that is “hangover tolerance” develops.

## 2. Methods

The relationship between hangover frequency and severity was analyzed using data from three independent studies. Study 1 was a survey by Penning et al. [11] among Dutch students that retrospectively assessed hangover frequency and severity. Study 2 was an international survey among young adults who recorded hangover severity on three different days and related these scores to hangover frequency. The retrospective assessment for multiple days is important as it has been suggested that hangover severity not only varies between drinkers but also within the same individual [2,12]. Using the average hangover severity score over the three days accounts for any intra-individual variation. Study 3 was a semi-naturalistic laboratory study with real-time assessments of hangover severity.

Study 1 was approved by the Institutional Review Board of the Utrecht Institute for Pharmaceutical Sciences of Utrecht University. No formal medical ethics approval was required to conduct this survey according to the Central Committee of Research Involving Human Subjects, The Netherlands. Study 2 received ethics approval from the Ethics Committee of the Faculty of Social and Behavioral Sciences of Utrecht University (FETC17-063). Study 3 also received ethics approval from the Ethics Committee of the Faculty of Social and Behavioral Sciences of Utrecht University. Study 3 was registered at www.clinicaltrials.gov (ClinicalTrials.gov Identifier: NCT01400204).

### 2.1. Study 1: Dutch Students Survey

Study 1 was a survey among Dutch students [11]. The survey comprised questions about drinking behavior, and the nature and severity of symptoms experienced during the past month latest hangover. Demographic data were collected (e.g., age, sex, height, and weight) and questions regarding usual drinking behavior were assessed, including a question on how many hangovers participants had experienced during the past month. The number of alcoholic drinks consumed for the evening before their past month latest hangover was recorded. The start and stop time of drinking alcohol was not measured in this study; therefore, the estimated blood alcohol concentration (eBAC) could not be computed. Subjects were asked to rate the severity of a list of 47 hangover symptoms they possibly experienced on their most recent hangover experience within the past month. From these, three outcome measures of overall hangover severity were computed. First, a modified version of the Hangover Symptoms Scale (HSS) [13] was compiled. This included the following 12 items; being tired, headache, nausea, vomiting, weakness, thirst, concentration problems, sensitivity to light, sweating, anxiety, depression, and shaking/shivering. For this study, the HSS item ‘trouble sleeping’ was omitted. The rationale for excluding this item was that it is not a true hangover symptom [14]. Trouble sleeping, if anything, is part of the cause, rather than the effect of hangover. As such, it may have an impact on the presence and severity of hangover symptoms [15]. The original HSS assessed the frequency of occurrence of hangover symptoms. To obtain a severity score, in our modified version, the severity of each item was rated on an 11-point scale from 0 (absent) to 10 (extremely severe). The mean score of the 12 items was used as a measure of overall hangover severity. Second, the Acute Hangover Scale (AHS) score was computed [16]. Third, the Alcohol Hangover Severity Scale (AHSS) score was computed [14]. Data from those subjects aged 18–30 who reported a past month alcohol hangover were included in the statistical analyses, which were conducted with SPSS (SPSS, version 25.0, SPSS Inc., Armonk, NY: IBM Corp). The analyses included correlating past month hangover frequency with overall hangover severity scores, using partial correlations, adjusting for the number of alcoholic drinks consumed.

### 2.2. Study 2: International Survey

An international survey was conducted in Nadi, Fiji among people working or on holiday. Subjects were approached to complete the survey at Wailoaloa Beach, Nadi, Fiji. The survey was anonymous, and participants did not receive an incentive for completing the survey. Potential participants were approached between 8 a.m. and 4 p.m. They were asked if they had consumed alcohol during the past three days. Those who were willing to participate and understood the English language were handed the survey. The investigator was present to address any queries with regards to language comprehension of the participants (who included international holidaymakers).

The survey collected demographic data such as gender, age, and country of citizenship. Usual weekly alcohol consumption (at home, before going to Fiji) was recorded, as well as information on how many day subjects were abroad from their home country, and how long they had been in Fiji.

Data regarding alcohol consumption and hangover severity were recorded for three consecutive days. Both the number of alcoholic drinks and the timeframe of consumption were recorded. The survey contained guidance about standard drinking sizes and how to convert, for example, bottles of wine into standardized alcohol units. Estimated blood alcohol concentration (eBAC) was computed, applying a modified Widmark formula [17]. On each test day, subjects reported their overall hangover severity on an 11-point scale ranging from 0 (absent) to 10 (extreme). Further questions asked about alcohol consumption when at home (before coming to Fiji), how many days per month they experienced a hangover, and how many times they had a hangover during their stay in Fiji.

Statistical analysis was performed using the SPSS statistical program (SPSS, version 25.0, SPSS Inc., Armonk, NY: IBM Corp). The participants who reported they did not have a hangover were excluded. Data from the three days were averaged to provide a more reliable average measure of alcohol intake and hangover severity. The hangover frequency at home and in Fiji was correlated with the average overall hangover severity score, using a partial correlation, adjusting for eBAC.

### 2.3. Study 3: Naturalistic Study

Students, aged 18 to 30 years old, were recruited at different locations (university canteen and colleges) of the university campus of Utrecht University, The Netherlands. The subjects remained anonymous, and after finalizing the study, they were paid 20 Euros for their participation. This naturalistic, observational study comprised two assessments: one the day after an evening of alcohol consumption, and the second assessment was made after an alcohol-free day. The investigators took steps to not influence the subjects’ drinking behavior or activities on the test days. There were no lifestyle or other guidelines imposed on the subjects, except that they were asked to complete the online survey between 2 p.m. and 10 p.m. The test days were scheduled one to three weeks apart, and the exact dates of the test days were chosen by the subjects themselves, depending on whether they choose to consume alcohol or not.

Demographic data were collected (e.g., age, sex, height, and weight) and questions regarding usual drinking behavior were assessed, including a question on how many hangovers participants had experienced during the past year. The Five-Shot questionnaire alcohol screening test was used to analyze general drinking behavior [18]. Personality was assessed with the Brief Symptom Inventory (BSI) [19] and the RT18 risk-taking questionnaire [20].

The BSI is a broad-used multidimensional symptom self-report inventory. It consists of 53 items; each rated on a five-point scale of distress from 1 (not-at-all) to 5 (extremely). The instrument is scored on nine symptom dimensions; somatization, obsession-compulsion, interpersonal sensitivity, depression, anxiety, hostility, phobic anxiety, paranoid ideation, and psychoticism. 

The RT-18 consists of 18 items that can be scored as ‘yes’ or ‘no’ [19]. Higher scores imply greater levels of risk-taking. Two subscales can be computed, namely ‘level of risk-taking behavior’ and ‘risk assessment’.

The Five-Shot questionnaire alcohol screening test was used to detect heavy drinking [18]. It is a short, self-report inventory, composed of two questions from the Alcohol Use Disorders Identification Test (AUDIT) [21,22] and three questions from the CAGE test [23]. A score of 2.5 or greater indicates possible alcohol misuse.

The number of alcoholic drinks consumed on the night prior to the test day was recorded, including the start and stop time of drinking alcohol. The estimated blood alcohol concentration (eBAC) was computed using a modified Widmark equation [17]. The modified Widmark equation takes into account the number of alcoholic drinks, relative body water volume, weight, gender, time taken to clear alcohol through metabolism. Participants were also asked how many alcoholic drinks they had consumed on this occasion, relative to a ‘regular’ drinking occasion. For each test day, subjects reported their overall hangover severity on an 11-point scale ranging from 0 (absent) to 10 (extreme). In addition, subjects rated their alcohol hangover via the modified 13-item version of the HSS [13], on which the severity of each item was rated on an 11-point scale from 0 (absent) to 10 (extremely severe).

Statistical analysis was performed using the SPSS statistical program (SPSS, version 25.0, SPSS Inc., Armonk, NY: IBM Corp). Subject reporting not having a hangover were excluded from the analysis. Past month hangover frequency was correlated with overall hangover severity scores, using partial correlations, adjusting for eBAC.

## 3. Results

### 3.1. Study 1: Dutch Students Survey

A total of 1410 participants completed the questionnaire, of which 56.1% (*n* = 791) had experienced at least one hangover during the last month. Their data were used for the analysis. The mean (SD) age of the sample was 20.4 (3.4) years, and 68.7% of the sample were women. The results of the study are summarized in Table 1.

Overall hangover severity, measured using the HSS, correlated significantly and positively with the frequency of past month hangovers (*r* = 0.188, *p* = 0.000). When adjusting for the number of alcoholic drinks consumed, the partial correlation remained significant (*r* = 0.126, *p* = 0.001).

Overall AHS hangover severity correlated significantly and positively with the frequency of past month hangovers (*r* = 0.145, *p* = 0.000). When adjusting for the number of alcoholic drinks consumed, the partial correlation remained significant (*r* = 0.125, *p* = 0.001).

Overall AHSS hangover severity correlated significantly and positively with the frequency of past month hangovers (*r* = 0.198, *p* = 0.000). When adjusting for the number of alcoholic drinks consumed, the partial correlation remained significant (*r* = 0.153, *p* = 0.001).

### 3.2. Study 2: International Survey

The international survey was completed by n = 333 subjects (145 men and 188 women). Subjects originated from 20 different countries, with most of them coming from the UK (34.6%), Australia (11%), France (9.4%), USA (8.7%), and New Zealand (7.1%). Their mean (± SD) age was 23.5 (± 4.2) years old, and they reported weekly alcohol consumption of 11.5 (± 10.9) drinks. They reported experiencing 3.9 (± 3.4) hangovers per month at home, and 4.8 (± 13.2) while at Fiji. There was no significant difference in hangover frequency reported at home or in Fiji (*t* = −1.26, *p* = 0.208). On average they were 78 (± 102) days abroad from their home country, of which the past 26 (± 26) days were spent at Fiji. In Fiji, alcohol consumption and hangover data were gathered for three consecutive days, and the results are summarized in Table 2. A partial correlation, adjusting for eBAC, revealed that the associations between hangover severity and hangover frequency at home (*r* = 0.197, *p* = 0.024) and hangover frequency at Fiji (*r* = 0.276, *p* = 0.001) remained significant.

### 3.3. Study 3: Naturalistic Study

A total of n = 99 subjects participated in the study. Subjects were excluded if they used drugs of abuse on the test days (n = 3) or alcohol on the control day (n = 3). N = 12 other subjects were excluded because they reported no hangover on the alcohol test day. N = 81 subjects were eligible for the present analysis, of which 36 (44.4%) were men. The subjects had a mean (SD) age of 21.2 (2.9) years old. The associations between drinking variables and hangover frequency and severity are summarized in Table 3.

**Figure 1:**
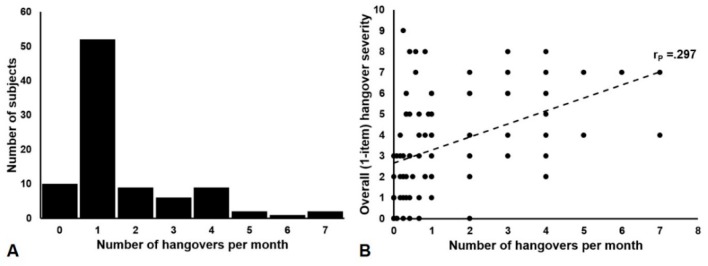
Distribution of the hangover frequency scores (**A**) and their association with the overall hangover severity (**B**). Note: a partial correlation (*r_P_*), adjusting for estimated blood alcohol concentration, was computed between the hangover frequency and severity.

As expected, hangover frequency was significantly correlated with a weekly number of alcoholic drinks consumed, the Five-Shot score, and the number of alcoholic drinks consumed, and eBAC on the evening before the hangover. Similarly, both HSS and the one-item hangover severity was significantly associated with the weekly number of alcoholic drinks consumed, the Five-Shot score, and the number of alcoholic drinks consumed, and the eBAC on the evening before the hangover. Figure 1 shows the distribution of hangover frequencies per month (Figure 1A) and its correlation with the one-item hangover severity (Figure 1B).

A positive and significant Spearman’s correlation was found between the hangover frequency and HSS hangover severity (*r* = 0.452, *p* = 0.000) and between hangover frequency and the one-item hangover severity (*r* = 0.529, *p* = 0.000). When conducting a partial correlation, adjusting for eBAC, the correlations between the hangover frequency and the severity remained significant and positive (HSS: *r* = 0.301, *p* = 0.004, 1-item: *r* = 0.297, *p* = 0.004). 

The partial correlation between the hangover frequency and the severity also remained significant when in addition to eBAC, was also corrected for the usual alcohol intake variables (weekly number of alcoholic drinks consumed and the Five-Shot score) (HSS: *r* = 0.297, *p* = 0.005, 1-item: *r* = 0.341, *p* = 0.001). When, in addition to eBAC, the correlation also adjusted for the hangover test day variables (number of alcoholic drinks consumed on the drinking occasion and the drinking duration), the correlation between the hangover frequency and severity also remained significant (HSS: *r* = 0.318, *p* = 0.002, 1-item: *r* = 0.283, *p* = 0.007).

### 3.4. Individual Hangover Symptoms

The severity of individual hangover symptoms and their frequency of being reported are summarized in Table 4. An exploratory analysis was conducted to identify individual hangover symptoms of which the reported severity was associated with hangover severity. Partial correlations, adjusting for eBAC, were computed, and a Bonferroni’s correction was applied to account for the multiple comparisons (*p*-values are considered statistically significant if *p* < 0.004). The analysis revealed that only the severity score of headache correlated significantly with hangover frequency (See Table 4).

### 3.5. Personality Characteristics

It is unlikely that the association between the hangover frequency and the severity could be explained by personality characteristics, including somatization. In Study 3, partial correlations, adjusting for eBAC and all BSI subscales, also revealed significant correlations between hangover frequency and severity (HSS: *r* = 0.217, *p* = 0.050, 1-item: *r* = 0.361, *p* = 0.000). Also, partial correlations, adjusting for eBAC and RT-18 risk-taking subscales, revealed significant correlations between hangover frequency and severity (HSS: *r* = 0.277, *p* = 0.008, 1-item: *r* = 0.278, *p* = 0.008).

### 3.6. Age

It could be argued that the hangover frequency is a proxy measure of age. As age progresses, the lifetime number of experienced hangovers increases. Also, research has suggested that the severity of hangover declines when age progresses [24], along with a steady reduction in the number of binge drinking days when age progresses [25]. To investigate the possible impact of age on the association between the hangover frequency and severity, the partial correlations in study 1, 2, and 3 were now corrected for both eBAC and age. It should be taken into account that the age range for the three studies, 18-30 years old, was relatively small. In study 1 the partial correlation, adjusting for the number of drinks consumed and age, between hangover frequency and hangover severity remained significant when assessed with either the HSS (*r* = 0.125, *p* = 0.001), AHS (*r* = 0.121, *p* = 0.002), or the AHSS (*r* = 0.151, *p* = 0.000). In study 2, the partial correlation, adjusting for eBAC and age, between hangover severity and hangover frequency at home or at Fiji remained significant (*r* = 0.195, *p* = 0.026, and *r* = 0.274, *p* = 0.002, respectively). In study 3, the partial correlation, adjusting for eBAC and age, between hangover frequency and hangover severity assessed with the HSS or the one-item hangover rating also remained significant (*r* = 0.286, *p* = 0.006, and *r* = 0.300, *p* = 0.004, respectively).

## 4. Discussion

Across studies, a significant and positive correlation was found between hangover frequency and severity, suggesting that when hangovers are experienced more frequently, their severity increases. These findings run counter to our prediction that tolerance develops to the effects of alcohol hangover. Therefore our hypothesis was not supported.

Our findings, are in contrast to those of the recent study by Köchling et al. [10], who reported that hangover frequency and severity are unrelated. The studies differed, however, in the methodologies to assess hangover frequency. Whereas Köchling et al. [10] used five relative crude frequency categories, in the current studies, the actual number of hangovers per month was calculated, which gives a more precise measure. Also, as opposed to our studies, Köchling et al. [10] used an unvalidated hangover scale to assess hangover severity, and an overall hangover severity rating was not obtained. Alternatively, one could argue that the study by Köchling et al. [10] was a controlled experiment, while our data was gathered via survey research and a study utilizing a less controlled naturalistic design. These methodological differences may account for the different outcomes of the study. Future research is, therefore, necessary to elucidate the exact nature of the relationship between hangover frequency and severity.

Our findings also contrast with the literature pertaining to acute intoxication, which suggests that, to some extent, tolerance develops to the acute effects of alcohol. This can be conceptualized as a rightward shift in the dose-response curve whereby repeated exposure to alcohol is manifested in two ways. The first is by individuals becoming less sensitive to the behavioral effects of the same dose of alcohol; the other is by requiring a greater amount of alcohol to achieve previous effects. 

The physiological evidence of this effect includes data showing that more frequent drinkers develop pharmacokinetic tolerance. This is illustrated by reports of lower breath alcohol concentration (BAC) to the same level of alcohol compared with less frequent drinkers [26]. Consistent with this finding, after consuming the same dose of alcohol, frequent drinkers may report fewer adverse sedative effects [27] and feel less intoxicated [28]. In addition, behavioral tolerance to the acute effects of alcohol may develop differentially, depending on the task domain [29]. Behavioral tolerance, i.e., the observation that greater experience with drinking to intoxication leads to less impaired cognitive and psychomotor performance, has been observed in several studies [29,30,31,32,33]. In this context, a recent study showed that drinkers with higher levels of performance maintenance and those who experienced less severe intoxication effects, self-administered higher dosages of intravenous alcohol and reached higher peak BACs than drinkers who were more sensitive to the effects of alcohol [34]. Finally, research has shown that the sensitivity to acute alcohol effects varies with age such that, across several domains, young drinkers are less sensitive than older drinkers to alcohol intoxication effects [35].

Taken together, there is evidence that more frequent consumption of greater alcohol quantities results in tolerance to the acute effects of alcohol. In contrast to the assumption that alcohol hangover would exhibit similar characteristics [7], the reduced sensitivity to acute alcohol effects is not reflected in lower sensitivity to hangover effects. Instead, the opposite was observed: the data suggest that with increased hangover frequency, the severity of hangovers becomes worse. In the context of the current findings, it appears that repeated exposure to alcohol differentially affects intoxication and hangover. That is, repeated exposure to alcohol was related to increases in the magnitude of the hangover effect. This leftward shift in the dose-response is characteristic of sensitization or reverse tolerance.

A strength of the current paper is that the results were consistent across several studies using different methodologies (surveys and naturalistic study). Different instruments were applied to assess overall hangover severity (one-item score, HSS, AHS, and AHSS, and aggregate severity scores over three days). Despite the variation in these measures, in all cases, a significant and positive association was found between hangover frequency and severity. Additionally, the sample sizes were sufficiently large to be confident about this outcome. This finding persists when adjusting for alcohol consumption variables (e.g., the amount of alcohol consumed and eBAC), age, or personality characteristics.

The current findings have several implications. Firstly, as no tolerance to hangover severity develops but rather the opposite, this may have consequences for the functional outcomes of the alcohol hangover. That is, behavioral effects may also further deteriorate in drinkers who exhibit more frequent hangovers. This is an important issue for further research, as reverse tolerance may have a significant impact on the magnitude of impairments seen in the hangover state on common daily activities such as driving a car [36,37].

Secondly, the findings suggest that alcohol hangovers do not act as a deterrent to further alcohol consumption. In fact, even though hangovers become worse with frequency, drinkers persist in consuming alcohol to levels that produce hangovers. Previous studies [38,39] also noted that experiencing hangovers does not have a relevant impact on future drinking behavior. One implication of this is that there may be value in investigating harm reduction strategies which act as ‘hangover treatments’ [40,41,42].

The studies also have limitations that should be addressed. The first one is the possibility of recall bias. The survey by Penning et al. [11] gathered data retrospectively. Therefore, hangover severity may not have been accurately recalled. However, the naturalistic study and International survey recorded hangover severity in real-time and showed similar results. Moreover, the observed correlation between hangover frequency and severity in real-time was of greater magnitude in the naturalistic study than the correlations observed when data was collected retrospectively. Secondly, hangover frequency was recorded via a single question. In future research, it could be considered to apply alternative methods such as the Time Line Follow Back approach [43] to reduce the possible impact of recall bias. Thirdly, hangover severity assessment may be biased, as this is a single measurement rather than an average over multiple drinking sessions. A single assessment may not accurately represent the typical hangover severity experienced by drinkers. However, the Fiji survey made hangover severity assessments on three consecutive days, and average scores were used for the analysis to account for intrapersonal differences and day-to-day fluctuations in alcohol consumption and hangover severity. Analysis of the Fiji dataset revealed similar results as the single timepoint survey and the naturalistic study. Thus, the observations cannot be regarded as coincidental, depending on the unique unknown features affecting hangover severity of one hangover occasion.

Fourthly, there is the issue of experimental control. None of the three datasets were drawn from controlled experiments with a set amount of alcohol consumed, nor were drink types standardized or controlled by the investigators. Therefore, hangover severity scores are influenced by several factors such as total amount of alcohol consumed, drinking duration, congener content, and eBAC. This may also explain in part why our findings differ from the controlled study conducted by Köchling et al. [10]. In the statistical analysis, the variability in drinking patterns was controlled by computing partial correlations, adjusting the observed associations between hangover frequency and severity for eBAC or the number of alcoholic drinks consumed.

Fifthly, the assessment of hangover frequency was done by simply asking subjects how many hangovers they had experienced per month, or during the past year. It is likely, however, that hangover frequency varies across a lifetime, along with periods of higher and lower alcohol intake. The current analysis did not take this variability into account as this data was not collected. There are alternative measures that could be applied, such as lifetime number of experienced hangovers that may be more informative in this regard and could be applied in future research. 

Sixthly, the age range of study participants, 18–30 years old, was small. This may have an impact on the relationship between hangover frequency and hangover severity. Future studies should, therefore, also include older-aged drinkers. Although longitudinal research showed that reported past year hangover frequency remains relatively stable from when assessed one year apart [6], Piasecki et al. [44] also followed student drinkers for 11 years and showed a steady decline in past year hangover frequency. Tolstrup et al. [24] also reported that during adulthood (18–65 years old) with increasing age, the frequency of hangovers gradually ameliorates. Although the title of the paper suggests otherwise, a closer look at the data revealed that they did not assess hangover severity. Instead, the frequency of experiencing nine hangover symptoms was assessed. The observation that the occurrence of hangovers declined with increasing age persisted after correction for the usual amount of alcohol intake, frequency of binge drinking, and the proportion of alcohol consumed with meals. There are various socioeconomic and cultural factors that may contribute to the reduction in hangover frequency when age progresses. For example, the start of job and family life responsibilities at the transition from student to adult life may reduce the frequency and quantity of alcohol consumption. Research has shown that the number of binge drinking days reduces with progressing age [25]. Hence, when age progresses, given the reduced amount of alcohol consumed per drinking session, there is a reduction of the opportunities to have a hangover per se. As stated above, the association between hangover frequency and age is complex, and there may be many moderating variables, which should be the subject of future research. For example, it has also been found that infrequent drinkers (14 or fewer days per month) consumed more alcohol on drinking days and were more frequently involved in binge drinking (13.4%) compared to frequent drinkers (4.3%) [45]. Taken together, future research on the association between hangover frequency and severity should take age into account as a moderating factor.

Finally, there is no validated, reliable assessment scale to determine the vulnerability and sensitivity of alcohol hangover. It should be the aim of future research to develop such a scale, to be able to, for example, select study subjects that are sensitive to hangover effects per se at a given number of alcoholic drinks, or to create homogenous research samples. The current findings can then be replicated in prospective studies or controlled experimental studies with standardized alcohol intake.

In addition to the limitations described above, future research should also elucidate the possible reasons for variability in the presence and severity of alcohol hangover. This is important because although a consistent positive association was shown between hangover frequency and hangover severity, correlational analysis does not imply causality. There may be other factors than hangover frequency that may be the actual cause of variability in hangover severity. Similarly, the regression analysis by Kochling et al. [10] cannot prove the absence of a causal relationship between hangover frequency and severity. The association between hangover frequency and severity is complex, and there may be many moderating variables. Future research should address biopsychological age-related factors that may impact the association between hangover frequency and severity, such as deterioration of liver function, psychological changes, motives for alcohol consumption, and cognitive decline.

Slutske et al. [13] found that only 13% of 1265 students reported having experienced no hangover symptoms during the past year. Most subjects reported 1–2 past year hangovers (27%) followed by 3–11 past year hangovers (34%). Hangover resistance is reported by around 25% of drinkers who reach BACs around 0.10–0.12% [46], but the percentage depends on the amount of alcohol consumed and at higher dosages significantly fewer drinkers claim to be hangover resistant [47,48,49]. 

Studies reported that subjects with a positive family history of alcoholism reported more frequent hangovers than drinkers with a negative family history of alcoholism [13,44,49]. In an experimental study, Span and Earlywine [50] found that subjects with a positive family history of alcoholism reported more severe hangovers than subjects with a negative family history of alcoholism.

Genetic profiling may elucidate why these differences exist. An Australian twin study revealed that 43% of hangover resistance could be explained by genetic influences [51]. Genetic factors accounted for 40% (men) to 45% (women) of variability in hangover frequency. A US twin study reported 55% heritability of the frequency of having hangovers [52]. In both studies, a close relationship was observed with genetic variability in the frequency of being intoxicated. The severity of hangovers is also influenced by genetic factors, for example by variability in alleles decoding for aldehyde dehydrogenase (ALDH2). For example, it has been found that Asian American students with ALDH2*2 alleles may experience more severe hangovers [53], and similar findings were reported for Japanese workers [54]. Unfortunately, in both studies, a direct relationship between hangover frequency and hangover severity was not assessed. Nevertheless, Wall et al. [53] suggested that tolerance develops to the risk of having hangovers, as they found that higher levels of usual alcohol intake were associated with increased hangover frequency and with reduced hangover severity. This suggestion is, however, not supported by the current findings.

## 5. Conclusions

A positive and significant association between hangover frequency and severity was found, which remained significant after correcting for alcohol intake. This finding suggests that hangovers become worse when they are experienced more often. Future research should further investigate this, and factors mediating the observed association, including its implications for alcohol prevention.

## Figures and Tables

**Table 1:** The association between drinking variables and the severity and frequency of hangovers.

Hangover	Mean (SD)	Frequency	Severity (AHS Score)	Severity (AHSS Score)	Severity (HSS Score)
Usual consumption					
weekly number of alcoholic drinks	16.8 (14.6)	*r* = 0.583, *p* = 0.000	*r* = 0.170, *p* = 0.000		*r* = 0.167, *p* = 0.000
number of past month hangovers	2.4 (2.2)	---	*r* = 0.145, *p* = 0.000		*r* = 0.188, *p* = 0.000
Hangover day					
number of alcoholic drinks	10.6 (5.9)	*r* = 0.329, *p* = 0.000	*r* = 0.164, *p* = 0.000	*r* = 0.211, *p* = 0.000	*r* = 0.196, *p* = 0.000
HSS hangover severity	3.1 (1.5)	*r* = 0.188, *p* = 0.000	*r* = 0.873, *p* = 0.000	*r* = 0.913, *p* = 0.000	---
AHSS hangover severity	3.0 (1.6)	*r* = 0.153, *p* = 0.000	*r* = 0.874, *p* = 0.000	---	*r* = 0.913, *p* = 0.000
AHS hangover severity	3.7 (1.7)	*r* = 0.145, *p* = 0.000	---	*r* = 0.188, *p* = 0.000	*r* = 0.873, *p* = 0.000

Note: Partial correlations, adjusting for the number of alcoholic drinks consumed before the hangover day, were computed to relate the hangover frequency with severity. For other associations, non-parametric (Spearman’s rho) correlations were computed.

**Table 2:** The association between drinking variables, hangover severity, and hangover frequency.

Hangover (3 Day Average)	Mean (SD)	Frequency at Home	Frequency at Fiji	Severity (1-Item)
Number of alcoholic drinks consumed	6.0 (5.2)	*r* = 0.188, *p* = 0.001	*r* = 0.228, *p* = 0.000	*r* = 0.504, *p* = 0.000
eBAC (%)	0.11 (0.1)	*r* = 0.216, *p* = 0.012	*r* = 0.250, *p* = 0.004	*r* = 0.454, *p* = 0.000
Drinking duration (h)	5.3 (3.5)	*r* = 0.062, *p* = 0.443	*r* = 0.197, *p* = 0.014	*r* = 0.294, *p* = 0.000
1-item hangover severity score	1.2 (1.5)	*r* = 0.309, *p* = 0.000	*r* = 0.468, *p* = 0.000	---

Note: data represent the average scores of three consecutive days of alcohol consumption and experiencing hangovers. Abbreviation: eBAC = estimated blood alcohol concentration.

**Table 3:** Association between drinking variables and severity and frequency of hangovers.

Hangover	Mean (SD)	Frequency	Severity (HSS Score)	Severity (1-Item)
Usual consumption				
weekly number of alcoholic drinks	8.4 (7.4)	*r* = 0.695, *p* = 0.000	*r* = 0.324, *p* = 0.002	*r* = 0.695, *p* = 0.000
the Five Shot score	2.6 (1.2)	*r* = 0.507, *p* = 0.000	*r* = 0.206, *p* = 0.048	*r* = 0.507, *p* = 0.000
Number of hangovers per month	1.4 (1.7)	---	*r* = 0.452, *p* = 0.000	*r* = 0.529, *p* = 0.000
Hangover day				
number of alcoholic drinks consumed	9.2 (4.6)	*r* = 0.452, *p* = 0.000	*r* = 0.413, *p* = 0.000	*r* = 0.452, *p* = 0.000
eBAC (%)	0.16 (0.1)	*r* = 0.416, *p* = 0.000	*r* = 0.421, *p* = 0.000	*r* = 0.416, *p* = 0.000
drinking duration (h)	6.3 (2.2)	*r* = 0.174, *p* = 0.095	*r* = 0.236, *p* = 0.023	*r* = 0.174, *p* = 0.095
HSS hangover severity score	2.3 (1.4)	*r* = 0.452, *p* = 0.000	---	*r* = 0.718, *p* = 0.000
1-item hangover severity	3.5 (2.5)	*r* = 0.529, *p* = 0.000	*r* = 0.718, *p* = 0.000	---

Note: Non-parametric (Spearman’s rho) correlations were computed. These are considered statistically significant if *p* < 0.004, after Bonferroni’s correction for multiple comparisons.

**Table 4:** The severity of individual hangover symptoms and past year’s hangover frequency.

Hangover Symptom	Mean (SD)	Frequency Reported	Correlation with Hangover Frequency
Being tired	5.6 (2.6)	97.8%	*r* = 0.059, *p* = 0.580
Thirst	4.7 (3.0)	90.3%	*r* = 0.176, *p* = 0.095
Weakness	4.1 (3.0)	83.9%	*r* = 0.238, *p* = 0.023
Concentration problems	3.6 (2.7)	81.7%	*r* = 0.242, *p* = 0.021
Headache	2.8 (3.2)	58.1%	*r* = 0.340, *p* = 0.001 *
Nausea	2.3 (2.8)	57.0%	*r* = 0.212, *p* = 0.044
Shaking, shivering	1.3 (2.2)	40.9%	*r* = 0.184, *p* = 0.081
Sleep problems	1.3 (2.1)	36.6%	*r* = 0.025, *p* = 0.811
Sensitivity to light	1.3 (2.2)	37.6%	*r* = 0.045, *p* = 0.672
Sweating	1.2 (2.2)	32.3%	*r* = 0.263, *p* = 0.012
Depression	0.6 (1.6)	15.1%	*r* = 0.182, *p* = 0.084
Anxiety	0.4 (1.3)	11.8%	*r* = 0.219, *p* = 0.037
Vomiting	0.4 (1.7)	6.5%	*r* = −0.047, *p* = 0.660

Note: Partial correlations, adjusted for eBAC, were computed. These are considered statistically significant if *p* < 0.004, after Bonferroni’s correction for multiple comparisons. Significance is indicated by *.
